# Measuring the Impact of Antiretroviral Therapy Roll-Out on Population Level Fertility in Three African Countries

**DOI:** 10.1371/journal.pone.0151877

**Published:** 2016-03-25

**Authors:** Milly Marston, Jessica Nakiyingi-Miiro, Victoria Hosegood, Tom Lutalo, Baltazar Mtenga, Basia Zaba, on behalf of the ALPHA network

**Affiliations:** 1 London School of Hygiene and Tropical Medicine, London, United Kingdom; 2 Medical Research Council, Entebbe, Uganda; 3 Africa Centre for Health and Population Studies, University of KwaZulu-Natal, Somkhele, South Africa; 4 Faculty of Medicine, Faculty of Social and Human Sciences, University of Southampton, Southampton, United Kingdom; 5 TAZAMA Project, National Institute of Medical Research, Mwanza, Tanzania; 6 Rakai Health Sciences Program, Kalisizo, Rakai, Uganda; Johns Hopkins University, UNITED STATES

## Abstract

**Background:**

UNAIDS official estimates of national HIV prevalence are based on trends observed in antenatal clinic surveillance, after adjustment for the reduced fertility of HIV positive women. Uptake of ART may impact on the fertility of HIV positive women, implying a need to re-estimate the adjustment factors used in these calculations. We analyse the effect of antiretroviral therapy (ART) provision on population-level fertility in Southern and East Africa, comparing trends in HIV infected women against the secular trends observed in uninfected women.

**Methods:**

We used fertility data from four community-based demographic and HIV surveillance sites: Kisesa (Tanzania), Masaka and Rakai (Uganda) and uMkhanyakude (South Africa). All births to women aged 15–44 years old were included in the analysis, classified by mother’s age and HIV status at time of birth, and ART availability in the community. Calendar time period of data availability relative to ART Introduction varied across the sites, from 5 years prior to ART roll-out, to 9 years after. Calendar time was classified according to ART availability, grouped into pre ART, ART introduction (available in at least one health facility serving study site) and ART available (available in all designated health facilities serving study site). We used Poisson regression to calculate age adjusted fertility rate ratios over time by HIV status, and investigated the interaction between ART period and HIV status to ascertain whether trends over time were different for HIV positive and negative women.

**Results:**

Age-adjusted fertility rates declined significantly over time for HIV negative women in all four studies. However HIV positives either had no change in fertility (Masaka, Rakai) or experienced a significant increase over the same period (Kisesa, uMkhanyakude). HIV positive fertility was significantly lower than negative in both the pre ART period (age adjusted fertility rate ratio (FRR) range 0.51 95%CI 0.42–0.61 to 0.73 95%CI 0.64–0.83) and when ART was widely available (FRR range 0.57 95%CI 0.52–0.62 to 0.83 95%CI 0.78–0.87), but the difference has narrowed. The interaction terms describing the difference in trends between HIV positives and negatives are generally significant.

**Conclusions:**

Differences in fertility between HIV positive and HIV negative women are narrowing over time as ART becomes more widely available in these communities. Routine adjustment of ANC data for estimating national HIV prevalence will need to allow for the impact of treatment.

## Background

There has been a rapid scale up in the provision of Antiretroviral therapy (ART) in Sub Saharan Africa in the last decade with more than 7.5 million people receiving treatment by the end of 2012[1] this comes hand in hand with increased access to HIV testing and therefore knowledge of HIV status. A large proportion of those with HIV in sub Saharan Africa are women of reproductive age who are routinely tested for HIV at antenatal care clinics (ANC) in order to try to prevent mother to child transmission and those testing positive are referred to clinics for treatment [2].

Official estimates of national HIV prevalence by UNAIDS are currently based on trends observed in antenatal clinic surveillance[3] and far more ANC data are becoming available due to routine reports from PMTCT programs. ANC prevalence trends are then adjusted to match prevalence levels estimated from national population surveys [4]. Part of this adjustment accounts for the reduced fertility of HIV positive women [5, 6], however increased access to care and treatment services and uptake of antiretroviral therapy may impact on the fertility of HIV positive women for biological and behavioural reasons, implying a need to re-estimate the adjustment factors used in these calculations.

There have been no longitudinal studies in Sub-Saharan Africa that have looked at the population level impact of ART on fertility. A few studies have measured fertility or incidence of pregnancy in women on ART [7–10] but these lack suitable comparators (HIV negative women in the same community) and may not be representative of all HIV positive women. A cross sectional comparison using Malawian Demographic and Health Survey data (DHS) found an increased probability of giving birth for HIV positive women relative to HIV negative women between 2004 and 2010 [11] which is attributed to the increase in access to mother to child transmission and ART services in Malawi. We analyse the effect of antiretroviral therapy provision on population-level fertility in four cohort studies in Southern and East Africa, comparing trends in HIV infected women against the secular trends observed in uninfected women.

## Methods

### Sites and setting

Fertility data that span the pre-ART era and the time of introduction and widespread use of ART are available from four community-based demographic and HIV surveillance sites: Kisesa (managed by the National Institute of Medical Research Mwanza in Northern Tanzania), Masaka (MRC/UVRI Uganda Research Unit on AIDS), Rakai (Rakai Health Sciences)–both in South-west Uganda, and uMkhanyukude (Africa Centre, in KwaZulu-Natal, South Africa). These sites belong to the network for analysing longitudinal population based data on HIV in Africa (ALPHA)[12, 13] and have been described in detail elsewhere [14–20].

### Data, HIV and ART provision

The ALPHA network standardises site-specific data to a common format to enable joint analysis. In brief, each study records demographic data including dates of birth (of mothers and infants). The studies also collect data on HIV status and provide dates of testing and test results for their populations. In Kisesa, Rakai, and uMkhanyakude, the HIV surveys were done separately to the demographic surveillance rounds, and data were linked afterwards using unique identifiers. In Masaka, HIV testing was done immediately after demographic surveillance rounds which were used to list those eligible for HIV testing. HIV testing took place in the home for all sites apart from Kisesa where temporary village clinics are used to which people are transported from their homes. Prior to the availability of antiretroviral therapy, testing protocols used informed consent without disclosure, so that participants did not learn the results of the HIV research tests, however with the advent of ART, sites began to offer full pre-test and post-test counselling to the participants during the data collection round. Participants are still not obliged take part in the counselling or to learn their results. In Rakai the samples taken in the home were tested at the field laboratory and then returned by a community based counsellor to those participants requesting the results.

The introduction and level of uptake of ART in the different studies varies. ART was introduced in the study areas between 2004 and 2005, when selected clinics were allowed to administer drugs and people were mobilised to make them aware of the new services. Further details of ART introduction and uptake are described elsewhere[21–24].

### Study Population

The population included in this analysis is women of reproductive age (15–44) living in the surveillance areas between the time point corresponding to five years before ART was introduced, to the last date for which data were available for each site up to 2015. Table 1 shows prevalence for each site in pre and post ART years for women aged 15–49.

**Table 1:** HIV prevalence of women aged 15–49 in Study sites.

Study Site, Round (years)	ART period	N	Prevalence	(95% CI)
Kisesa 4 (2003–2004)	Pre ART	3369	5.11	(4.36–5.85)
Kisesa 6 (2010–2010)	Post ART	2445	7.08	(6.06–8.09)
Manicaland 3 (2003–2005)	Pre ART	8273	20.45	(19.58–21.32)
Manicaland 5 (2009–2011)	Post ART	6150	17.54	(16.59–18.50)
Masaka 14 (2002–2003)	Pre ART	2361	14.06	(12.66–15.46)
Masaka 16 (2004–2005)	Post ART	2485	17.99	(16.48–19.50)
Rakai 9 (2002–2003)	Pre ART	3708	13.65	(12.54–14.75)
Rakai 12 (2006–2008)	Post ART	4507	14.98	(13.93–16.02)
uMkhanyakude 1 (2002–2005)	Pre ART	6533	27.2	(26.12–28.28)
uMkhanyakude 4 (2007–2008)	Post ART	3604	27.58	(26.12–29.04)

All live births to women aged 15–44 years old while under observation in the study were included in the analysis, classified by mother’s age, area of residence and HIV status at time of the birth, and by ART availability in the community. We did not include 45–49 year olds to create the standard fertility analysis grouping of 15–49 years as there were very few births to HIV positive women at this age.

All sites are predominately rural, though most contain areas with local markets, health and education facilities. Area of residence is classified differently for each site. The Masaka DSS is divided into two areas: old villages where surveillance began in 1989; and new villages where surveillance began in November 1999. In Kisesa sub-villages are classified according to their distance from the small trading centre on the main road. In Rakai the peri urban group comprises trading towns, villages along secondary roads and fishing communities and the rural category are communities beyond those located along secondary roads. uMkhanyakude is predominantly rural but also includes an small town and peri-urban densely populated areas.

### Measures

HIV Status was classified as negative, positive and unknown. Negative person time was defined as the time between first testing negative and last testing negative, also included in negative time was a site specific time following the last negative test, this was allocated according to the HIV incidence rates in the sites, the cut off for post negative time was taken as the time at which the cumulated probability of becoming infected following the last test reached 5%. This cut-off point was five years in Kisesa, Masaka and Rakai and two years in uMkhanyakude. HIV positive time was all the person time after the first positive test. The sero conversion interval was calculated as the mid point between first positive and last negative test and positive and negative time was assigned accordingly. Interval censoring was invoked if the midpoint of the sero conversion interval was longer than the site specific post negative time–in that case only the post negative time was assigned to negative and one year pre positive time was assigned to positive, the remainder of the interval was designated unknown. The composition of the unknown group over time changes due to the different ways HIV positive and HIV negative time is allocated and is also affected by participation changes in testing [25, 26] therefore we do not present results from this group. No pressure was put on participants to receive their HIV status as part of research survey procedures, therefore we would not expect a link between participation in testing and fertility.

**Fig 1:**
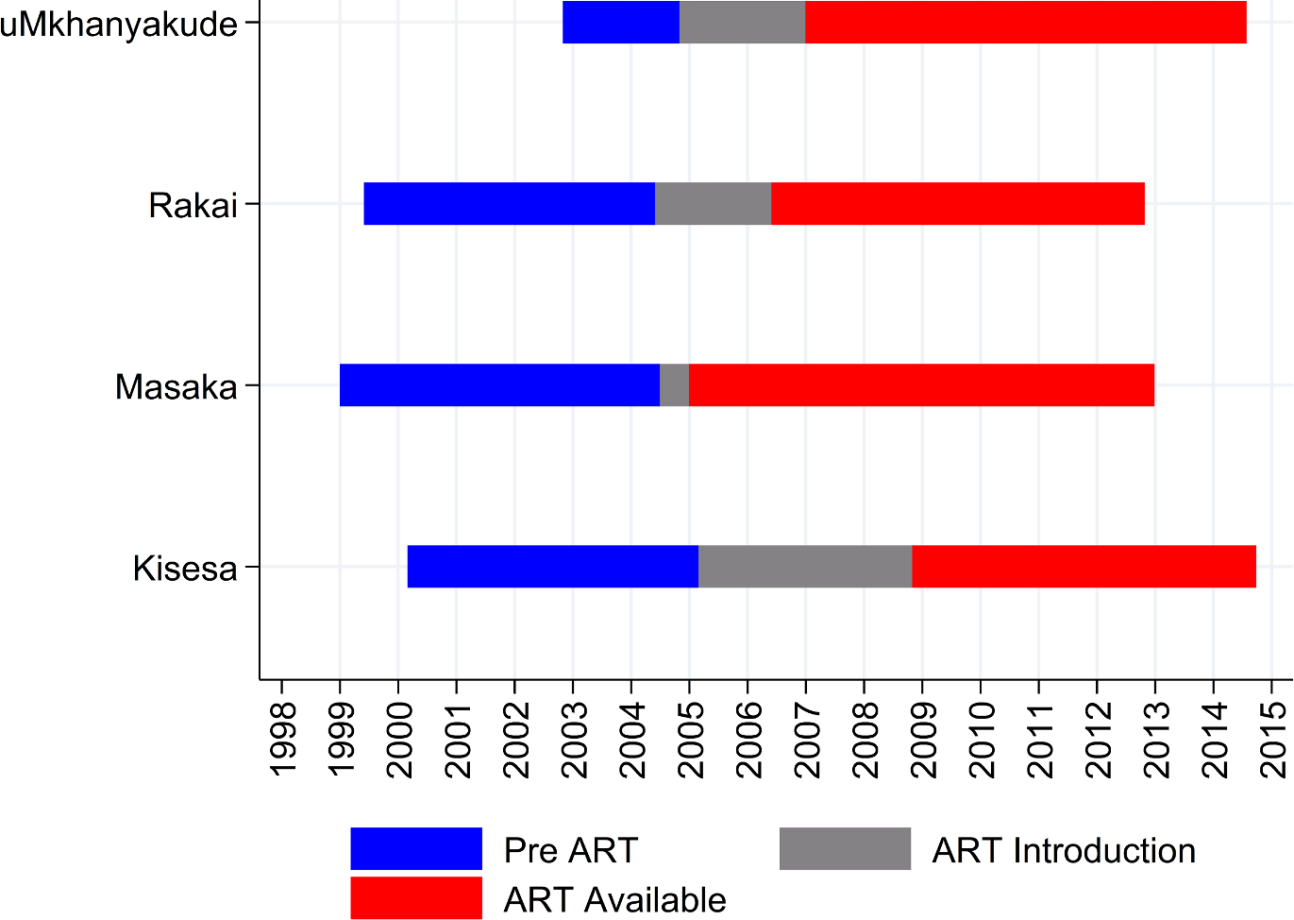
Dates of ART Periods included in the analysis.

Calendar time period of data availability relative to ART introduction varied across the sites (Fig 1), from 5 years prior to ART roll-out, to 9 years after. Calendar time was classified according to ART availability, grouped into pre ART, ART Introduction (introduced in at least one of the health facilities serving the community) and ART available (available in all the health facilities serving the community that were designated as ART providers according to national guidelines). We limit results presented for the short ART roll out period to just the age specific rates, as this time period is relatively short and is different in each site depending on the speed and nature of the roll out therefore tells us little about general patterns and trends.

### Analysis

We used Poisson regression to calculate age adjusted fertility rate ratios over time by HIV status, and investigated the interaction between ART period and HIV status to ascertain whether trends over time were different for HIV positive and negative women. We adjust for age and area of residence in this analysis to control for any changes in the composition of the study site that may have occurred between the pre and post ART periods. The analysis was performed separately for each site and pooled across sites where appropriate, the pooled results were adjusted by study site.

At ages under 20 HIV infection and fertility are highly correlated as many teenagers will not yet have had sex, and only those who have had sex are at risk of becoming pregnant or acquiring HIV. Therefore fertility in the 15–19 year old age group is almost always higher in HIV positive women [6]. We therefore stratify our analysis to look at 15–19 year olds and 20–44 year olds separately.

### Ethics statements

Each of the six sites contributing data to the pooled analysis has received ethical clearance from the appropriate local ethics review bodies, and from the corresponding Institutional Review Boards for studies which had collaborating partnerships with Northern Universities.

#### uMkhanyakude

Annually re-certified ethics permission for the Africa Centre DSS and nested individual HIV surveillance among consenting adults is obtained from the Biomedical Research Ethics Committee at the Nelson Mandela School of Medicine, University of KwaZulu-Natal. Detailed written informed consent obtained for participation in the HIV surveillance.

#### Kisesa

Ethical approval for each survey round of the Kisesa cohort study granted by the Tanzanian Medical Research Coordinating Committee and the Ethics Committee of the London School of Hygiene and Tropical Medicine. Prior to 2006, verbal consent was obtained directly from all study participants (aged 15 and over), due to low literacy rates among the study population. Consent was witnessed and documented for each study participant by a member of the sero-survey team or senior person from the community. From 2006 onward, consent was again obtained directly from all study participants, however a written consent option was introduced, for those able to provide this and written parental consent for those under 18 years was also obtained.

#### Masaka

The MRC DSS is approved by the Uganda Virus Research Institute (UVRI) Science and Ethics Committee (SEC) and the Uganda National Council of Science and Technology (UNCST). Study participants provided written consent to participate in all parts of the study.

#### Rakai

The Rakai Community Cohort Survey is approved by the UVRI SEC and UNCST. Literate participants provided written consent while those unable to read or write had a witness sign on their behalf. Those unable to read or write used a thumbprint to document consent and a witness would also sign as evidence that the consent had been read to the participant who had understood and consented to participate.

## Results

Overall crude and age specific fertility rates fell or remained the same between the pre and post ART periods for all sites (Tables 2–5). Classifying the crude fertility rates by HIV status shows that overall fertility declined in the HIV negative but remained more or less the same or increased slightly for the HIV positive (For the HIV positive in Rakai this is true if we exclude 15–19 year olds). Age specific rates by HIV status follow this general trend for all sites apart from slight increases in HIV negative 25–34 year olds in Masaka, 35–39 year olds in Rakai and 15–19 year olds in uMkhanyakude. Overall fertility is higher in the rural areas for all sites.

**Table 2:** Fertility of women aged 15–44 years old, by calendar time period and stratified by individual HIV status for Kisesa.

	All			HIV-Negative			HIV-Positive		
	Births	Person Years	Rate /1000	Births	Person Years	Rate /1000	Births	Person Years	Rate /1000
**Kisesa**									
** ART Period**									
** **Pre ART	5253	26.2	200.1	4108	17.6	233.1	132	1.0	126.9
** **ART Introduction	3715	19.5	190.6	2763	12.6	218.4	106	0.8	132.0
** **ART available	5895	37.2	158.3	2924	15.4	190.3	195	1.4	144.2
** Age**									
** ***15–19*									
** **Pre ART	846	6.2	136.6	584	3.6	160.2	12	0.1	197.6
** **ART Introduction	518	4.8	108.3	308	2.9	107.3	8	0.0	232.3
** **ART available	792	9.5	83.6	335	3.6	93.3	4	0.0	81.5
** ***20–24*									
** **Pre ART	1461	5.4	270.8	1117	3.4	325.8	23	0.2	144.7
** **ART Introduction	952	3.8	250.7	714	2.3	309.2	9	0.1	97.5
** **ART available	1475	7.2	205.1	637	2.7	237.1	24	0.1	193.6
** ***25–29*									
** **Pre ART	1294	4.9	264.1	1002	3.2	311.7	44	0.3	157.6
** **ART Introduction	897	3.3	270.1	676	2.1	315.3	34	0.2	202.7
** **ART available	1402	6.3	223.9	693	2.4	285.1	40	0.2	185.5
** ***30–34*									
** **Pre ART	877	4.0	221.3	705	2.8	254.3	38	0.3	143.7
** **ART Introduction	746	3.1	237.7	563	2.1	272.0	32	0.2	133.3
** **ART available	1131	5.6	200.5	621	2.4	254.7	66	0.3	197.1
** ***35–39*									
** **Pre ART	569	3.3	173.6	510	2.6	198.8	13	0.2	80.4
** **ART Introduction	426	2.4	180.9	356	1.7	212.8	16	0.2	102.2
** **ART available	805	5.0	162.5	454	2.3	201.2	55	0.4	131.9
** ***40–44*									
** **Pre ART	206	2.5	81.8	190	2.0	95.0	2	0.1	17.3
** **ART Introduction	176	2.1	83.9	146	1.6	92.3	7	0.1	62.6
** **ART available	290	3.7	78.1	184	2.0	93.7	6	0.2	28.3
** Residence**									
** ***Rural*									
** **Pre ART	3125	13.4	233.2	2564	10.1	254.2	71	0.4	171.0
** **ART Introduction	2295	9.9	232.0	1805	7.2	252.4	54	0.3	162.7
** **ART available	3453	17.9	193.0	1932	8.9	215.9	103	0.6	164.3
** ***Peri Urban/Urban*									
** **Pre ART	2128	12.8	165.7	1544	7.5	204.8	61	0.6	97.6
** **ART Introduction	1420	9.6	147.9	958	5.5	174.2	52	0.5	110.4
** **ART available	2442	19.3	126.3	992	6.4	154.6	92	0.7	126.8

**Table 3:** Fertility of women aged 15–44 years old, by calendar time period and stratified by individual HIV status for Masaka.

	All			HIV-Negative			HIV-Positive		
	Births	Person Years	Rate /1000	Births	Person Years	Rate /1000	Births	Person Years	Rate /1000
**Masaka**									
** ART Period**									
** **Pre ART	2524	14.8	170.1	2285	12.8	179.2	117	1.0	115.2
** **ART Introduction	510	3.3	153.6	466	2.8	164.0	21	0.3	83.8
** **ART available	4185	28.0	149.7	3672	23.3	157.4	281	2.3	119.7
** Age**									
** ***15–19*									
** **Pre ART	490	4.6	107.0	448	4.2	106.9	14	0.1	205.3
** **ART Introduction	101	1.0	105.3	91	0.9	102.3	2	0.0	112.2
** **ART available	665	8.5	78.5	593	7.6	77.6	25	0.2	159.4
** ***20–24*									
** **Pre ART	766	2.9	261.3	701	2.5	277.6	32	0.2	185.3
** **ART Introduction	155	0.6	245.1	145	0.5	266.8	3	0.0	77.3
** **ART available	1115	4.9	226.6	1005	4.1	242.6	58	0.3	186.8
** ***25–29*									
** **Pre ART	562	2.4	231.9	497	2.0	252.0	38	0.3	136.5
** **ART Introduction	126	0.5	229.9	115	0.4	260.9	6	0.1	101.3
** **ART available	1012	4.3	237.1	884	3.5	256.1	71	0.4	163.7
** ***30–34*									
** **Pre ART	357	1.9	192.4	316	1.5	211.9	20	0.2	88.8
** **ART Introduction	69	0.4	154.4	63	0.4	175.6	4	0.1	69.3
** **ART available	786	4.0	195.3	680	3.2	215.4	71	0.5	133.5
** ***35–39*									
** **Pre ART	263	1.7	152.8	241	1.4	168.4	11	0.2	62.7
** **ART Introduction	38	0.4	98.4	32	0.3	100.4	5	0.0	117.7
** **ART available	466	3.3	139.6	387	2.6	149.6	47	0.5	92.1
** ***40–44*									
** **Pre ART	86	1.3	64.9	82	1.1	71.7	2	0.1	20.9
** **ART Introduction	21	0.3	60.4	20	0.3	68.9	1	0.0	29.0
** **ART available	141	2.9	48.1	123	2.3	52.6	9	0.4	22.2
** Residence**									
** ***Original Study Villages*									
** **Pre ART	1989	11.3	175.5	1820	9.9	183.6	81	0.7	116.5
** **ART Introduction	382	2.4	158.1	347	2.1	164.8	16	0.2	97.8
** **ART available	3003	19.5	153.7	2675	16.6	161.4	182	1.5	121.4
** ***New villages*									
** **Pre ART	535	3.5	152.7	465	2.8	163.8	36	0.3	112.4
** **ART Introduction	128	0.9	141.6	119	0.7	161.6	5	0.1	57.4
** **ART available	1182	8.4	140.5	997	6.8	147.6	99	0.8	116.7

**Table 4:** Fertility of women aged 15–44 years old, by calendar time period and stratified by individual HIV status for Rakai.

	All			HIV-Negative			HIV-Positive		
	Births	Person Years	Rate /1000	Births	Person Years	Rate /1000	Births	Person Years	Rate /1000
**Rakai**									
** ART Period**									
** **Pre ART	6598	51.8	127.3	5805	35.9	161.8	495	5.5	90.2
** **ART Introduction	2598	23.1	112.5	2304	15.5	148.5	201	2.3	88.6
** **ART available	6634	68.7	96.6	5736	42.8	134.0	547	6.9	79.3
** Age**									
** ***15–19*									
** **Pre ART	1220	13.4	91.3	1119	8.1	137.3	38	0.3	131.0
** **ART Introduction	310	5.7	54.8	273	3.0	90.7	7	0.1	83.9
** **ART available	674	17.4	38.8	601	9.2	65.2	17	0.2	90.8
** ***20–24*									
** **Pre ART	2320	12.8	181.4	2090	9.6	217.9	137	1.1	127.4
** **ART Introduction	872	5.4	161.6	799	3.8	208.7	50	0.4	129.5
** **ART available	1953	14.8	131.8	1651	9.0	183.0	125	0.9	139.0
** ***25–29*									
** **Pre ART	1713	10.3	166.7	1460	7.3	199.5	183	1.6	116.7
** **ART Introduction	791	4.8	165.2	693	3.5	199.0	80	0.6	133.4
** **ART available	1992	13.7	145.9	1641	8.6	190.0	178	1.6	112.6
** ***30–34*									
** **Pre ART	830	6.4	128.8	684	4.4	156.5	96	1.3	74.6
** **ART Introduction	425	3.4	124.9	369	2.4	155.8	40	0.6	65.0
** **ART available	1309	10.9	119.7	1077	6.8	158.5	131	1.7	75.4
** ***35–39*									
** **Pre ART	382	4.8	79.0	331	3.4	96.4	36	0.8	45.4
** **ART Introduction	155	2.0	76.0	130	1.5	88.9	19	0.3	55.1
** **ART available	572	7.1	80.3	458	4.3	106.1	65	1.3	50.8
** ***40–44*									
** **Pre ART	133	4.1	32.4	121	3.0	40.2	5	0.5	10.5
** **ART Introduction	45	1.8	24.9	40	1.4	29.3	5	0.2	21.0
** **ART available	134	4.8	28.1	116	2.9	40.3	7	0.8	8.4
** Residence**									
** ***Rural*									
** **Pre ART	5483	41.2	133.2	4872	29.3	166.4	390	4.2	91.9
** **ART Introduction	2200	18.2	121.1	1979	12.6	157.3	157	1.7	91.7
** **ART available	5156	49.7	103.7	4459	31.7	140.8	400	4.7	85.9
** ***Peri Urban/Urban*									
** **Pre ART	1115	10.7	104.5	933	6.6	141.4	105	1.2	84.3
** **ART Introduction	398	4.9	80.7	325	2.9	110.7	44	0.6	79.1
** **ART available	1401	18.5	75.7	1019	8.9	114.7	118	1.8	65.1

**Table 5:** Fertility of women aged 15–44 years old, by calendar time period and stratified by individual HIV status for uMkhanyakude.

	All			HIV-Negative			HIV-Positive		
	Births	Person Years	Rate /1000	Births	Person Years	Rate /1000	Births	Person Years	Rate /1000
**uMkhanyakude**									
** ART Period**									
** **Pre ART	3337	31.8	104.8	1550	14.0	110.7	297	3.0	98.3
** **ART Introduction	3899	33.7	115.7	1887	15.3	123.6	581	4.9	119.1
** **ART available	11601	119.5	97.0	4741	41.0	115.7	2264	23.7	95.4
** Age**									
** ***15–19*									
** **Pre ART	794	9.3	85.2	485	5.9	82.6	45	0.4	120.7
** **ART Introduction	1019	9.8	104.2	664	6.4	103.4	85	0.4	196.8
** **ART available	2836	32.8	86.4	1639	17.3	94.5	217	1.4	159.3
** ***20–24*									
** **Pre ART	1014	6.6	154.5	435	2.4	182.2	95	0.7	138.7
** **ART Introduction	1262	7.4	169.6	589	3.2	182.4	211	1.1	184.9
** **ART available	3749	25.2	149.0	1631	9.3	175.4	587	4.2	140.2
** ***25–29*									
** **Pre ART	653	4.8	135.3	209	1.3	167.0	77	0.7	116.3
** **ART Introduction	689	4.9	139.5	228	1.3	170.4	133	1.0	129.4
** **ART available	2398	20.3	118.2	661	4.5	145.5	680	5.6	122.0
** ***30–34*									
** **Pre ART	491	4.1	121.0	206	1.2	168.9	47	0.5	86.7
** **ART Introduction	505	4.2	119.5	184	1.2	158.3	95	0.9	103.1
** **ART available	1487	15.9	93.8	397	3.0	132.4	483	5.1	94.7
** ***35–39*									
** **Pre ART	284	3.6	79.9	154	1.5	104.6	27	0.4	64.8
** **ART Introduction	301	3.7	80.6	148	1.4	106.0	43	0.8	57.0
** **ART available	880	13.5	65.2	305	3.1	99.0	240	4.2	56.8
** ***40–44*									
** **Pre ART	101	3.5	28.7	61	1.8	34.0	6	0.3	17.4
** **ART Introduction	123	3.6	34.3	74	1.7	42.9	14	0.6	23.3
** **ART available	251	11.9	21.1	108	3.7	29.2	57	3.3	17.3
** Residence**									
** ***Rural*									
** **Pre ART	2069	18.9	109.8	1076	9.6	112.2	165	1.6	100.3
** **ART Introduction	2382	19.8	120.2	1288	10.3	124.9	335	2.7	123.9
** **ART available	6955	66.2	105.1	3220	26.8	120.4	1190	12.2	97.7
** ***Peri Urban/Urban*									
** **Pre ART	1192	12.2	97.5	452	4.2	107.1	126	1.3	95.0
** **ART Introduction	1387	12.6	110.0	548	4.6	118.9	234	2.0	114.8
** **ART available	4476	48.4	92.5	1486	13.7	108.3	1048	10.8	97.1

**Fig 2:**
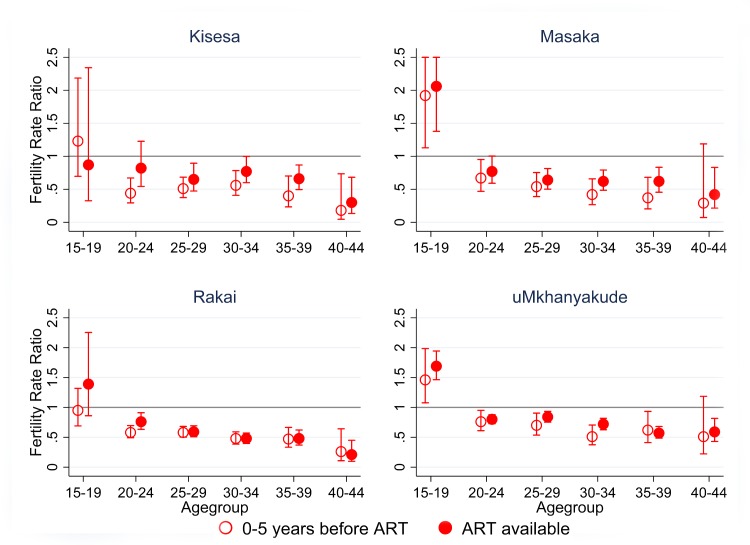
Unadjusted fertility rate ratios by age and ART period comparing positives to negatives.

Fertility rates in HIV positive women are consistently lower than those of HIV negative women apart from the youngest age group of 15–19 years olds (Fig 2). For all sites apart from uMkhanyakude fertility rates in HIV positive women aged over 20 are around half those of the negatives in the pre ART period and 0.73 (95%CI 0.64–0.83) times the negative rates in uMkhanyakude. In the post ART period overall the differences between positive and negative are smaller, with rate ratios ranging from 0.57 (95%CI 0.52–0.62) in Rakai to 0.83 (95%CI 0.78–0.87) in uMkhanyakude. In both periods fertility differences between positive and negative women become greater as age increases.

For all women aged 15–44 years old, age and residence adjusted fertility rates declined significantly over time, driven by the significant decline in fertility among HIV negative women in all four studies (Table 6). For HIV positive women in Kisesa and Masaka there is a fertility increase of borderline significance of 1.21(95%CI 0.99–1.49) and 1.16(95%CI 0.96–1.41) respectively, and no change in uMkhanyakude and Rakai. The interaction term between HIV Status and ART period was significant for all sites apart from uMkhanyakude showing that the changes in fertility over the two ART periods are significantly different for HIV positive and HIV negative women. Excluding 15–19 year olds (Table 7) whose fertility rates are determined largely by patterns of sexual debut, yields an overall reduction in fertility in HIV negative women in all sites and increases among HIV positive women in Kisesa and Masaka with rate ratios of 1.29 (95%CI 1.04–1.59) and 1.21 (95%CI 0.99–1.47) respectively, and no change in uMkhanyakude and Rakai. In this narrower age range the interaction terms between HIV Status and ART period were significant for all sites apart from Rakai.

**Table 6:** Fertility Rate Ratio (FRR) for 15–44 year olds comparing ART period with pre ART adjusted for by age and residence.

	ART Period	HIV Negative		HIV Positive		Interaction p-value*
		FRR	(95% CI)	FRR	(95% CI)	
*Kisesa*						
	Pre-ART	1		1		
	ART Available	0.84	(0.81–0.88)	1.21	(0.99–1.49)	0.001
*Masaka*						
	Pre-ART	1		1		
	ART Available	0.90	(0.86–0.94)	1.16	(0.96–1.41)	0.010
*uMkhanyakude*						
	Pre-ART	1		1		
	ART Available	0.98	(0.93–1.03)	1.04	(0.92–1.16)	0.363
*Rakai*						
	Pre-ART	1		1		
	ART Available	0.84	(0.81–0.86)	0.96	(0.86–1.08)	0.020

*Interaction between ART period and HIV status

**Table 7:** Fertility Rate Ratio (FRR) for 20–44 year olds comparing ART period with pre ART adjusted for by age and residence.

	ART Period	HIV Negative		HIV Positive		Interaction p-value*
		FRR	(95% CI)	FRR	(95% CI)	
*Kisesa*						
	Pre-ART	1		1		
	ART Available	0.89	(0.86–0.93)	1.29	(1.04–1.59)	0.001
*Masaka*						
	Pre-ART	1		1.00		
	ART Available	0.94	(0.90–0.99)	1.21	(0.99–1.47)	0.016
*uMkhanyakude*						
	Pre-ART	1		1		
	ART Available	0.90	(0.84–0.95)	1.04	(0.92–1.17)	0.033
*Rakai*						
	Pre-ART	1		1		
	ART Available	0.93	(0.90–0.96)	1.00	(0.89–1.12)	0.252

*Interaction between ART period and HIV status

The data for 20–44 year olds were pooled for the comparison of the period when ART was available with the pre ART period, giving overall rate ratios of 0.9 (95%CI 0.89–0.92) for HIV negative women and 1.08 (95%CI 1.01–1.16) for HIV positive women adjusted for age, residence and study site, with a significant interaction (p<0.001) between HIV status and ART period. Focussing on 15–19 year olds (Table 8), there has been a significant reduction in fertility for HIV negative women in all sites apart from uMkhanyakude where the relative increase was 1.11 (95%CI 1.01–1.22). The confidence intervals for the rate ratios for the HIV positive are very large, so no real pattern can be determined.

**Table 8:** Fertility Rate Ratio (FRR) for 15–19 year olds comparing ART period with pre ART adjusted for by age and residence.

	ART Period	HIV Negative		HIV Positive		Interaction p-value*
		FRR	(95% CI)	FRR	(95% CI)	
*Kisesa*						
	Pre-ART	1		1		
	ART Available	0.56	(0.49–0.64)	0.40	(0.14–1.12)	0.524
*Masaka*						
	Pre-ART	1		1		
	ART Available	0.75	(0.67–0.85)	0.73	(0.41–1.32)	0.936
*uMkhanyakude*						
	Pre-ART	1		1		
	ART Available	1.11	(1.01–1.22)	1.32	(0.97–1.79)	0.278
*Rakai*						
	Pre-ART	1		1		
	ART Available	0.49	(0.44–0.53)	0.66	(0.38–1.13)	0.277

*Interaction between ART period and HIV stat

For the two sites with data available (Kisesa and Masaka) in the 5–10 years prior to ART there was no evidence of any interaction between HIV status and period when comparing the periods 0–5 years and 5–10 years prior to ART (Not shown).

Person years with unknown HIV status were lowest in Masaka at 7.2% in the pre ART period and 8.2% in the post ART period, in Rakai they were 20.2% and 27.7%, Kisesa 28.9% and 52.0%, uMkhanyakude 46.5% and 38.3% respectively. The HIV status unknown category includes the unclassified post negative time intervals and time before the first HIV test.

## Discussion

This analysis uses community based cohort studies to look at the population impact of ART on fertility. We have shown that changes in fertility have been different in HIV positive women compared to the HIV negative over the pre and post ART Period—representing a discontinuity since the pre ART era. This would indicate that the introduction of ART is narrowing the gap in fertility rates between the HIV positive and negative. These results are similar to those found in the cross sectional study using the Malawi DHS [11] which showed a decrease over two surveys in the relative difference in fertility comparing the HIV positive and HIV negative at the time of the survey. Since our longitudinal data can accurately measure HIV status at the time of birth these results are a strong affirmation of the cross-sectional findings.

Fertility dynamics, HIV and changes due to ART are complex and can be both biological and behavioural. Earlier studies showed that HIV positive women with further disease progression have lower fertility than the uninfected and those more recently infected [9, 10, 27]. Women with HIV also have increased risk of spontaneous abortion and still birth [28] which lower their fertility. It is possible that the improved health of women on ART increases their fecundity although one study found an increase in still births for HIV positive women on ART compared to those not on ART[29].

Relationship dynamics may also change: studies from the pre ART era have shown an increased risk of widowhood and marital dissolution for HIV positive women and low rates of remarriage [30, 31], therefore decreasing their chances to bear more children. In the era of ART the risk of widowhood will decrease and marital dissolution rates may change leading to more opportunity for childbearing.

Fertility intentions are likely also to change, HIV positive women are more likely to report desiring fewer births than those uninfected[32] but some studies that compared fertility intentions of HIV positive women on treatment to those not on treatment found an increase in desire for children with increasing duration on ART [7, 8, 33]. It is unclear whether these intentions translate into actual increases in fertility. A cross sectional study from a perinatal HIV Research Unit in Soweto found no difference in fertility intentions between those on ART or not[34]–however all participants were attending the HIV clinic so according to the authors, the intentions of those not yet on treatment may have been shaped by the knowledge that ART was available when needed. A multicounty HIV care and treatment program cohort study in sub-Saharan Africa reported HIV positive women on treatment having 1.74(95%CI 1.19–2.54) higher incidence of pregnancy than those not on treatment[9]. The study was unable to determine the factors underlying the results with both biological and behavioural factors being possible.

This analysis shows the extent of changes in fertility trends at the population level which is important for modellers and policy makers. It does not tell us how much of the change is attributable to biological factors directly associated with improved health of those receiving ART, psychological changes that alter fertility intentions, or social changes in marital dynamics and stigma. It is important to note that in the pre ART era most people in these studies would not have known their HIV status so reasons for low fertility in the HIV positives would not include a conscious desire for fewer children motivated by knowledge of status. These topics need further analysis with individual linkage to clinic data to classify time on treatment, to investigate biological factors, and more detailed demographic and behavioural background characteristics.

Differences in fertility between HIV positive and HIV negative women are narrowing over time as ART becomes more widely available in these communities. Routine adjustment of ANC data for estimating national HIV prevalence will need to allow for the impact of treatment. Given the profound differences between fertility rate ratios and trends in infected and uninfected women under 20 with those aged 20 and over, it would be useful to classify ANC data on HIV prevalence by age, reporting separately on those under 20.
